# Pityriasis Rosea With Multiple Herald Patches Resulting in a V-shaped Pattern and a Christmas Tree Distribution

**DOI:** 10.7759/cureus.52052

**Published:** 2024-01-10

**Authors:** Kazumasa Oya, Yoshiyuki Ishii, Keisuke Anju, Toshifumi Nomura

**Affiliations:** 1 Dermatology, University of Tsukuba, Tsukuba, JPN

**Keywords:** human herpesvirus, christmas-tree distribution, v-shaped pattern, herald patches, pityriasis rosea

## Abstract

Pityriasis rosea (PR), a benign and self-limiting skin disorder, typically manifests as a single initial lesion known as the herald patch. The herald patch is commonly followed by the development of secondary erythematous papules and plaques, aligning with Langer's lines to form a specific distribution pattern, resembling a Christmas tree on the back and a V-shaped pattern on the upper chest. Therefore, diagnosing PR may not be difficult based on its typical clinical presentation. In contrast, cases of atypical PR presentation have been reported, encompassing several differential diagnoses. Here, we present a case with multiple herald patches that needed differentiation from ringworm, syphilis, and erythema annular centrifugum. Subsequently, our case was diagnosed with PR, as the patches formed a V-shaped pattern and a Christmas-tree distribution.

## Introduction

Pityriasis rosea (PR) is a benign, self-limiting skin disorder characterized by a single initial papule that enlarges within several days, followed by the subsequent development of small lesions [1,2]. The prevalence of PR is estimated to be 0.21% [3]. It is most prevalent in the 18-25 age group, with its prevalence decreasing with increasing age [3]. Some studies have demonstrated an association of PR with a female predominance [3,4], while others yielded different results [5,6]. In addition to a higher prevalence during the winter season [4], prodromal symptoms similar to those experienced with viral infections (such as malaise, nausea, loss of appetite, and upper respiratory symptoms) have been reported in approximately 25-71% of cases [5-8]. Furthermore, human herpesvirus 6 (HHV-6) and human herpesvirus 7 (HHV-7) activation is observed in patients with PR [2,8,9], suggesting that viral infections and/or reactivation leading to abnormal immune reactions may trigger PR [8]. However, further research is required to determine the exact relationship between infectious agents and PR [10,11]. The cutaneous manifestation of PR usually begins as a single macule or papule on the trunk or neck, enlarging over a few days to form a 2 to 10-cm diameter with central scaling or a collarette-like border, known as a herald patch [2,9,11]. After the appearance of the herald patch, small papules subsequently emerge and align along the cleavage lines to form a V-shaped pattern on the upper chest and a Christmas-tree distribution on the back [2,9,11]. While several variants of PR have been reported [1], the clinical features and progression of PR with multiple herald patches remain unclear. In this report, we present a case of PR with multiple herald patches forming a V-shaped pattern and a distribution resembling a Christmas tree.

## Case presentation

**Figure 1:**
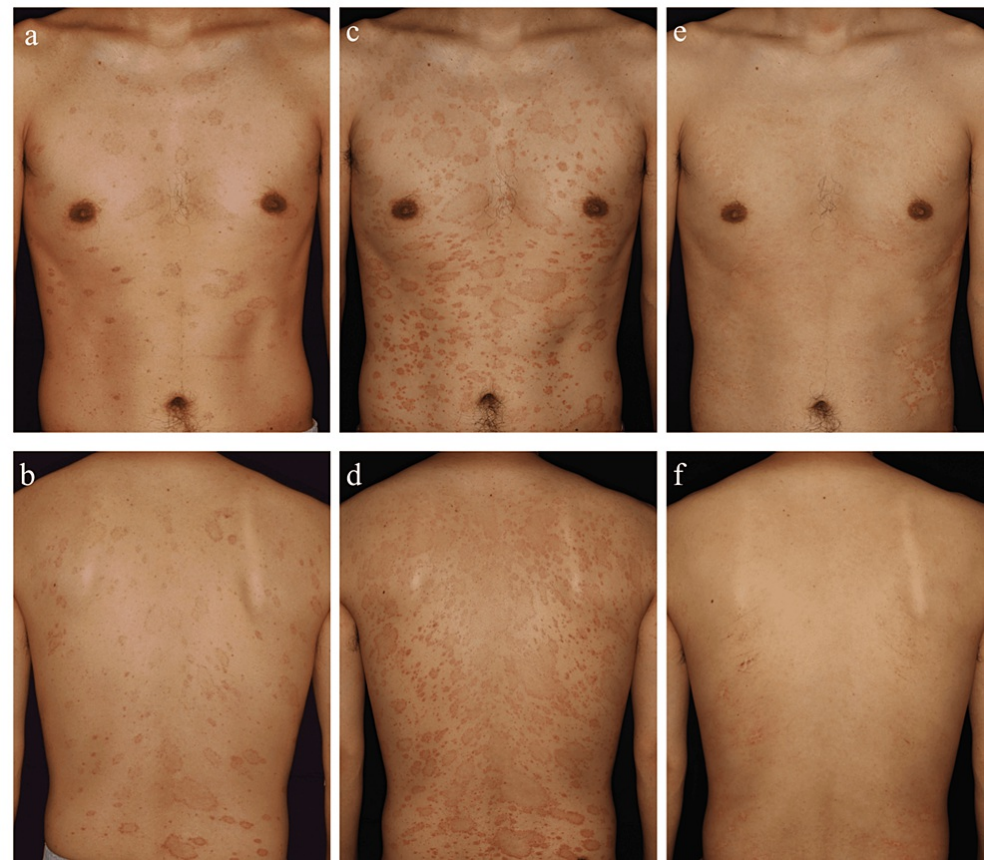
Clinical presentation The photograph at presentation showed oval and annular erythematous papules on his chest, abdomen (a), and back (b). One week after presentation, the lesions showed a V-shape pattern on his upper chest (c) and a Christmas-tree pattern on his back (d). Three weeks after the presentation, the lesions on his chest, abdomen (e), and back (f) improved.

A 32-year-old man presented with multiple annular erythematous lesions. He had no medical history or prodromal illness such as gastrointestinal or respiratory symptoms. Four weeks before the presentation, the lesions appeared around his trunk, and he experienced pruritus. Betamethasone butyrate propionate prescribed in another clinic was ineffective, necessitating referral to our dermatology department. Physical examination revealed oval and annular scaly papular erythematous patches on his trunk and extremities (Figure 1a, 1b). Laboratory testing revealed negative results of rapid plasma regain (RPR) and Treponema pallidum haemagglutination (TPHA) against Treponema pallidum. Potassium hydroxide examination of scrapings from the lesions revealed no fungal elements. A biopsy from the erythematous area revealed perivascular lymphocytic infiltrates and interface dermatitis. No HHV-6 and/or HHV-7 DNA was detected in the patient’s blood by real-time polymerase chain reaction. The lesions were exacerbated and enlarged, forming a V-shaped pattern on his upper trunk and a Christmas-tree configuration on his back one week after the initial visit (Figure 1c, 1d), leading to a diagnosis of PR. Continuous use of topical steroids resulted in improvement in the lesions within two weeks (Figure 1e, 1f). Based on the clinical course, the diagnosis of PR was established.

## Discussion

The herald patch commonly develops as a single lesion [1,2]; thus, the number of cases developing multiple forms of the herald patches is limited [12-14]. Mayfield et al. reported a case involving a school-going boy who had two herald patches on his left scapular region and multiple papules on his trunk [12]. Owing to the absence of itching, no treatment was prescribed [12]. Zawar et al. described the case of a nine-year-old boy with pruritic herald patches on his abdomen, thighs, and groin [13]. These herald patches appeared following the remission of fever, malaise, and sore throat, followed by the development of multiple scaly plaques on the posterior aspect of the trunk [13]. These lesions improved with the application of topical petroleum jelly and oral desloratadine, with mild post-inflammatory hypopigmentation [13]. Singh et al. reported the case of a 10-year-old girl who developed multiple asymptomatic herald patches without prodromal symptoms [14].

The initial application of topical steroids did not effectively prevent the spread of skin lesions in our case; however, continued use subsequently led to improvement. One possible explanation for this enlargement of lesions may be a robust disease magnitude at his first presentation beyond the responsiveness of short-duration steroid treatment. Given the immunosuppressive properties of topical steroids, continued treatment might have led to improvement. Another potential explanation is that PR naturally resolves without drug therapy around approximately eight weeks of onset [8,11], suggesting the possibility that lesions in our case may have cleared without the need for topical steroid treatment. While no consistent evidence supports the efficacy of topical steroid treatment for PR [15], topical steroids may be considered as a treatment option, particularly for patients with pruritic lesions, as observed in our case, given the anti-inflammatory effects of topical steroids. [1,2,15].

In the clinical course of PR, several days to weeks after the appearance of the herald patch, additional lesions manifest symmetrically on the trunk and proximal extremities [1,2]. These lesions gradually align along the skin's cleavage lines, forming a V-shaped pattern on the upper chest and a Christmas-tree distribution on the back [1,2]. V-shaped signs and a Christmas-tree distribution rash are recognized as distinct features of PR; however, cases that exhibit the coexistence of these two findings are limited. Our case is unique in that multiple round, oval erythematous papules, which were herald patches, were aligned in Langer's lines across the entire body, forming a coexistence of a V-shaped pattern with a Christmas-tree distribution. In addition, while a few studies mention the development of multiple herald patches, a comprehensive portrayal of the entire clinical progression of PR, encompassing the development of multiple herald patches to the subsequent emergence of V-shaped signs and a Christmas-tree distribution rash, along with the remission of the lesions, is lacking in the literature. Our paper addresses this gap by presenting the complete spectrum in detail, clearly illustrating individual skin lesions.

The herald patch and secondary lesions of PR share histological characteristics [16], characterized by hyperplasia of the epidermis, focal spongiosis, and focal parakeratosis with lymphocyte infiltration around vessels, as well as red blood cell extravasation [2,8,15]. These pathological features are classified as superficial perivascular dermatitis. Furthermore, histological findings of PR may manifest basal cell vacuolization [17], a feature of interface dermatitis. Consistent with these studies, histopathological analysis in our case revealed perivascular lymphocytic infiltrates and interface dermatitis. As these pathological findings are not specific to PR [2,17], the pathological analysis plays a supplementary role in diagnosing PR [8]. Therefore, the presence of multiple annular lesions in our case led to several differential diagnoses. One potential diagnosis in our case was ringworm, which can present with multiple well-demarcated scaly patches or plaques with distinct borders [18]. However, a potassium hydroxide examination did not reveal hyphae, and the lesions improved with topical steroid treatment, ruling out this diagnosis. Another possible diagnosis was syphilis, which may exhibit annular erythema. However, laboratory tests, RPR and THPA, ruled out syphilis. Another consideration was erythema annulare centrifugum (EAC), characterized by clear erythematous borders with scaling [19]. In our case, the herald patches comprised papular-erythematous lesions, which did not resemble EAC. Furthermore, the development of the V-shaped pattern with a Christmas-tree distribution over several days suggested that our case was more indicative of PR than EAC.

## Conclusions

PR is a common disease; its cutaneous manifestation typically begins with a single herald patch, followed by the subsequent development of eruptions forming a V-shaped pattern and a Christmas tree distribution. Therefore, specific tests are not mandatory for the diagnosis of PR with a typical presentation. However, the atypical appearance of herald patches, such as multiple herald patches, can pose a challenge for diagnosis. Collectively, our case underscores the significance of recognizing the atypical appearance of the herald patch for providing effective patient care.
